# Epidemiology, Phylogenetic Divergence, and Differential Pathogenicity of Feline Respiratory *Mycoplasma* in China

**DOI:** 10.1155/tbed/8765199

**Published:** 2026-06-02

**Authors:** Zijun Ye, Congrong Wang, Quanhui Yan, Weihui Li, Mingjun Ye, Yan Zhang, Luying Li, Qiuyan Li, Shengbo Cao

**Affiliations:** ^1^ National Key Laboratory of Agricultural Microbiology, College of Veterinary Medicine, Huazhong Agricultural University, Wuhan, Hubei, 430070, China, hzau.edu.cn; ^2^ Hubei Hongshan Laboratory, Wuhan, Hubei, 430070, China, hzau.edu.cn; ^3^ Frontiers Science Center for Animal Breeding and Sustainable Production, Huazhong Agricultural University, Wuhan, Hubei, 430070, China, hzau.edu.cn; ^4^ Wuhan Keqian Biology Co., Ltd., Wuhan, Hubei, 430000, China

**Keywords:** antimicrobial resistance, cross-protection analysis, epidemiology, *Mycoplasma felis*, virulence

## Abstract

*Mycoplasma felis* (*M. felis*) is a wall‐less pathogen known to induce respiratory diseases in felines. Current research on respiratory *M. felis* infections is scant, lacking established typing system and nationwide epidemiological survey. This study investigated the prevalence of *M. felis* in feline respiratory swabs (*n* = 4329) across China from 2022 to 2024. Coinfection profiles involving *M. felis*, feline herpesvirus (FHV), feline calicivirus (FCV), *Chlamydia felis* (*C. felis*), and *Bordetella bronchiseptica* (*Bb*) were assessed in 382 swab samples from cats with respiratory disease. Fifteen representative strains were isolated and characterized to provide insights for disease prevention and vaccine development. Phylogenetic analysis of these isolates revealed two novel clades: the Myco clade (exhibiting classical “fried‐egg” colonies) and the Morella clade (forming “mulberry‐like” microcolonies requiring supplemented media), alongside *M. gateae*–affiliated strains. Noteworthy strain‐specific metabolic adaptations were observed. Antimicrobial susceptibility testing revealed varying macrolide susceptibility (Morella > *gateae* > Myco), consistent fluoroquinolone sensitivity, and emerging doxycycline resistance. In vitro virulence was strongly associated with hydrogen peroxide (H_2_O_2_)production, with Morella‐type *M. felis*‐8 and Myco‐type *M. felis*‐28 showing the highest yields. In vivo challenges in cats validated differential pathogenicity: *M. felis*‐28 induced severe respiratory symptoms and lung histopathology (inflammatory infiltration and tissue disruption), while *M. felis*‐8 predominantly induced conjunctival injury. Cross‐protection analysis indicated limited antigenic conservation, with high autoreactivity (titer = 1:16) in strains such as *M. felis*‐47, but minimal cross‐reactivity (titer ≤ 1:2) among genetically distinct strains. These findings establish a novel diagnostic framework for distinguishing pathogenic clades, facilitating targeted therapeutic interventions and guiding rational vaccine development strategies for *M. felis*.

## 1. Introduction

*Mycoplasmas* represent significant pathogenic and commensal microorganisms isolated from diverse animal species [1]. As members of the class *Mollicutes* (which contains ~200 known species), *Mycoplasmas* are the smallest known prokaryotes. Unlike most bacteria, they lack a cell wall and are recognized as the smallest independently replicating life forms [2].

Feline *Mycoplasmas* are classified into two major types based on the site of infection: hemotropic *Mycoplasmas* and respiratory *Mycoplasmas* [2, 3]. Hemotropic *Mycoplasmas*, formerly known as *Haemobartonella felis*, are obligate erythrocyte parasites that can cause *hemolytic* anemia. Clinical manifestations include pale mucous membranes, jaundice, and lethargy [4, 5]. Respiratory *Mycoplasmas* are significant pathogens in feline upper respiratory tract disease (URTD), conjunctival, and pulmonary infections. Conjunctival colonization typically causes acute or chronic conjunctivitis, presenting with eyelid erythema and purulent ocular discharge [6]. Respiratory infections are characterized by chronic sinusitis (accounting for 15%–30% of cases) and pneumonia, with persistent sneezing, mucopurulent nasal discharge, and dyspnea [7]. These symptoms are often similar to those seen in viral respiratory infections, such as feline herpesvirus (FHV) and feline calicivirus (FCV), leading to challenges in clinical differentiation [8].

This research focuses on the two principal feline respiratory *Mycoplasma* species, *M. felis* and *M. gateae*, first isolated from cats in 1967 [9]. *Mycoplasma felis* can directly or indirectly cause respiratory infections in cats, and it has been reported in Japan that *M. felis* can also be isolated from other species, such as horses, indicating its potential for cross‐species transmission [10]. *Mycoplasma gateae* is an opportunistic pathogen with clinical symptoms similar to those of *M. felis*. Its pathogenicity is relatively weak when it acts alone [11], it typically contributes to disease in conjunction with other pathogens (e.g., viruses and bacteria) or as a secondary infection, often co‐occurring with *M. felis* or other pathogens [12]. The fastidious nature of *M. felis* complicates in vitro cultivation, limiting research into its pathogenicity and antimicrobial resistance profiles [13]. Hydrogen peroxide (H_2_O_2_), a key virulence factor in *Mycoplasma*s, is generated as a by‐product of glycerol metabolism and can induce host cell membrane damage, leading to tissue inflammation [14]. Although its pathogenic role has been established in various *Mycoplasma* species, including *M. hyopneumoniae* [13, 15, 16], the capacity of *M. felis* to produce H_2_O_2_ and its association with virulence remain poorly understood [13].

In recent decades, several countries have conducted epidemiological investigations into feline *Mycoplasma* infections to varying extents. According to data from the Australian Companion Animal Disease Surveillance System (2013–2015), among 3126 sampled cats, 569 were diagnosed with URTD, of which 46% (262 cases) tested positive for *M*. *felis* [17]. In France, 19 out of 55 cats with respiratory diseases admitted to the Centre Hospitalier Vétérinaire Fregis were positive for feline *Mycoplasmas* [18]. A 2020 report from the United States revealed that among 24 kittens exhibiting clinical signs of acute upper respiratory disease, eight (33%) tested positive for *M. felis* in ocular, oral, and pharyngeal swabs, with a positivity rate second only to FCV (54%) [6].

In China, although nationwide epidemiological data on feline *Mycoplasmas* remain lacking, multiple regional investigations have been conducted in recent years. A study in Wuhan involving 1158 cats with upper respiratory tract infection reported an *M. felis* positivity rate of 26.9% (311/1158), ranking second among the detected pathogens [19]. Surveillance of 374 urban stray cats in Shanghai revealed an *M. felis* positivity rate of 18.72% (70/374), the highest among all respiratory pathogens tested [20]. Another study in Kunshan, based on 458 samples from cats with respiratory infections, detected *M. felis* in 15.5% of cases [21]. Furthermore, a screening conducted by Chen et al. [22] in Guangxi Province, encompassing 396 cats, identified *M. felis* as the most prevalent pathogen associated with feline URTD in that region, with a positivity rate of 24.75%. These regional findings indicate that *M. felis* has reached considerable prevalence in certain areas of China; however, its infection status at the national level remains systematically uncharacterized.

The 2025 China Pet Industry White Paper indicates that the number of cats in China has rapidly increased in recent years, especially in the postpandemic era, with the number of stray cats increasing by 19% [23], accelerating pathogen transmission. Furthermore, the absence of a cell wall in *Mycoplasmas* confers intrinsic resistance to β‐lactam antibiotics, thereby impeding effective management strategies for *feline Mycoplasma* infections [24]. Reports from Southern China show an annual increase in cases of respiratory diseases caused by *M. felis* and *M. gateae*. However, systematic investigations into the prevalence, strain‐specific characteristics, and epidemiological distribution of *feline Mycoplasmas* across China remain scarce [8, 12].

While *M*.*felis* is a globally distributed pathogen, key aspects such as its pathogenic mechanisms, correlation with clinical signs, and epidemiological features remain incompletely understood and require further study [20]. This research has established the molecular epidemiological dataset for respiratory *Mycoplasmas* in China, isolated and identified new pathogenic strains, and elucidated key biological characteristics, thus, providing scientific evidence to optimize prevention strategies and support vaccine development.

## 2. Materials and Methods

### 2.1. Mycoplasma Liquid and Solid Media

For liquid broth, dissolve 21.0 g PPLO broth powder, 5.0 g glucose, and 1.0 mL 1% phenol red in 850 mL ddH_2_O with stirring. Adjust pH to 7.6–7.8 with 1 mol/L NaOH, bring volume to 900 mL, and sterilize at 116°C for 20 min. After cooling to approximately 60°C, add 1.0 mL penicillin (100,000 IU/mL), 1.0 mL 1% *w*/*v* NAD, and 100 mL porcine serum (10% final volume). Store at 2–8°C.

For solid agar, supplement the above formulation with 15.0 g agar prior to sterilization. After adding heat‐sensitive components, pour 15 mL per sterile plate and store at 2–8°C.

### 2.2. Epidemiological Sampling and Pathogen Isolation/Identification

Researchers employed a nationwide sampling strategy to obtain a representative epidemiological dataset. Sterile polyester swabs were used to collect oropharyngeal, nasal, and conjunctival samples from cats in three distinct settings (veterinary hospitals, catteries, and stray cat colonies) across 23 provinces in China. To ensure the randomness and representativeness of the data, sampling was conducted without preselection based on health status, encompassing both clinically symptomatic and asymptomatic individuals. The presence of clinical signs (e.g., sneezing, ocular discharge, and dyspnea) was systematically recorded by local collaborators or our research team at the time of collection, with samples being either collected on‐site or transported via cold chain to our laboratory.

Immediately after collection, swabs were placed in phosphate‐buffered saline (PBS) for preservation and transportation. Upon arrival at the laboratory, all samples were initially screened using PCR to detect five common feline respiratory pathogens: *M. felis*, FHV, FCV, *Chlamydia felis* (*C. felis*), and *Bordetella bronchiseptica* (*Bb*; Table 1) [25, 26]. The purpose of this initial multipathogen screening was to establish a comprehensive infection profile and to assess the coinfection status among these pathogens. Meanwhile, this study divided China into seven geographical regions to compare the number of positive respiratory swabs and positivity rates between healthy cats and cats with respiratory disease in different areas. The regional divisions are as follows: North China (Beijing, Hebei, and Shanxi); Northeast China (Jilin, Liaoning, and Heilongjiang); East China (Shanghai, Jiangsu, Zhejiang, Anhui, Fujian, Shandong, and Jiangxi); Central China (Hunan, Hubei, and Henan); South China (Guangdong and Guangxi Zhuang Autonomous Region); Southwest China (Chongqing, Sichuan, Yunnan, and Guizhou); Northwest China (Shaanxi and Xinjiang Uygur Autonomous Region).

**Table 1:** The primers designed for amplifying the genomes of feline respiratory pathogens.

Primer numbering	Primer sequences (5′ → 3′)	Fragment sizes (bp)
*M. felis*‐16‐23S‐F	ACACCATGGGAGCTGGTAAT	478
*M. felis*‐16‐23S‐R	GTTCATCGACTTTCAGACCCAAGGCAT	
*M. felis*‐16S‐F	AGAGTTTGATCCTGGCTCAG	1478
*M. felis*‐16S‐R	TACGGCTACCTTGTTACGACTT	
FHV‐F	GACGTGGTGAATTATCAGC	288
FHV‐R	CAACTAGATTTCCACCAGGA	
FCV‐F	GTTGACCCTTACTCATACAC	136
FCV‐R	CCCTGGGGTTAGGCGC	
*C.felis*‐F	TGGGACGATTGAGCGTAATG	600
*C.felis*‐R	AATCAATGCCGGTAGTTCTG	
*Bb*‐F	TGGCGCCTGCCCTATC	237
*Bb*‐R	AGGCTCCCAAGAGAGAAA	

For PCR detection, swab samples were vortexed in 1 mL of PBS. An aliquot of 200 μL supernatant was collected for genomic DNA extraction using a commercial spin column kit (Kexin Biotechnology, Wuhan, China). Each PCR run included a positive control (a known *M. felis*‐positive sample provided by the Microbiology Laboratory, Huazhong Agricultural University) and a negative control. Results were considered valid only when both controls yielded expected outcomes. Samples that tested positive (targeting the conserved *16S-23S rRNA* gene region, expected amplicon size: 478 bp) for *M. felis* were centrifuged at 3000 rpm for 10 min. The supernatant was then inoculated onto *Mycoplasma*‐specific solid agar plates and incubated at 37°C for 3–7 days. Following incubation, characteristic “fried‐egg” colonies were aseptically picked and transferred into *Mycoplasma* liquid broth until a color change of the pH indicator was observed. To ensure clonal purity, three consecutive purification cycles were performed, alternating between subculturing single colonies on solid agar plates and streaking liquid cultures onto fresh solid agar plates. The identity and genetic homogeneity of the final purified isolates were confirmed by Sanger sequencing of the *16S rRNA* gene.

### 2.3. Bioinformatic and Phylogenetic Statistical Analyses

Fifteen *M. felis* strains isolated in this study and 64 *Mycoplasma 16S rRNA* gene sequences from diverse species retrieved from NCBI were aligned for phylogenetic analysis. Sequence comparisons were performed using the MegAlign program within the Lasergene 7.0 software suite. Phylogenetic trees were constructed in MEGA 7.0 employing the neighbor‐joining method based on the Kimura 2‐parameter model, with bootstrap values calculated from 1000 replicates [27]. Statistical analyses were conducted using Chiplot software [28].

### 2.4. Microscopic Examination Techniques for *Mycoplasma felis*

*Mycoplasma felis* was inoculated onto PPLO agar plates containing 10% serum and 0.002% phenol red, and typical “fried egg‐like” colonies were observed using a stereomicroscope at 10x magnification. Colony morphology was documented with a Moticonnect digital microscope camera. Thin smears of *M. felis* culture were prepared, air‐dried, and heat‐fixed for Dienes staining [29]. *Mycoplasma felis* culture suspension was uniformly spread onto glass slides, air‐dried at ambient temperature, then overlaid with dienes staining solution and incubated for 1–2 min. After discarding the stain, slides were washed twice with PBS (1 min per wash), thoroughly dried by absorbent paper, and examined under microscopy. All isolated *M. felis* strains underwent purification via sucrose gradient centrifugation before preparing EM samples on copper grids. Sucrose density gradient centrifugation was performed as follows: a continuous density gradient was established by sequentially overlaying 2.5 mL each of 20%, 35%, 50%, and 65% sucrose solutions into a centrifuge tube. The concentrated bacterial suspension was carefully layered onto the top of the gradient and centrifuged at 15,000 rpm for 1 h at 4°C. Following centrifugation, distinct bands were visualized under illumination and carefully collected from the gradient interfaces. The fraction exhibiting the highest bacterial concentration was collected, diluted to 10 mL with PBS, and centrifuged at 12,000 rpm for 30 min at 4°C. This washing step was repeated three times. The final pellet was resuspended and transferred to a 1.5 mL centrifuge tube, then stored at −80°C until further use [30]. *Mycoplasma felis* images were captured using a Talos L120C TEM transmission electron microscope leased from the Wuhan Institute of Virology, Chinese Academy of Sciences.

### 2.5. Colorimetric Assay for *Mycoplasma* Metabolic Activity

To standardize the inoculum, a batch of *M. felis* culture at logarithmic growth phase was homogenized, aliquoted, and lyophilized for preservation. For each experiment, one vial of lyophilized powder was reconstituted, and its concentration was determined using the color‐changing unit (CCU) method (e.g., the stock solution was measured at 10^8.0^ CCU/mL). The reconstituted suspension was then diluted with PBS to 10^3.0^ CCU/mL as the working inoculum for subsequent liquid culture inoculation.

The experimental procedure comprised two stages: (1) Growth curve monitoring period: The *M. felis* isolate was cultured in liquid medium for 3 days, with sampling every 12 h for CCU determination and pH measurement. (2) CCU endpoint determination period: Each sample collected was serially diluted and incubated on solid medium for 7 days to allow colony formation, enabling retrospective calculation of CCU values corresponding to each time point. For pH assessment, measurements were performed using a calibrated portable pH electrode pen.

CCU quantification was based on the metabolic activity of *M. felis*, determined by serial dilution to identify the highest dilution capable of inducing a color change (1 CCU corresponds to the *Mycoplasma* quantity at this dilution). In 96‐well plates, 180 μL of *Mycoplasma* medium was dispensed into each well. The first well received 20 μL of bacterial suspension (10^−1^ dilution), which was mixed thoroughly before transferring 20 μL to the next well (10^−2^ dilution). This serial dilution was repeated through 10^−10^, with three replicate wells per dilution. Controls included uninoculated medium as negative control. All plates were incubated at 37°C under microaerophilic conditions (5% CO_2_) for 7 days. The CCU endpoint was defined as the highest dilution showing consistent color change for three consecutive days (e.g., if the 10^−8^ well changed color, while 10^−9^ remained unchanged, the CCU was recorded as 10^8^/mL).

### 2.6. MIC Testing of *Mycoplasma felis* Isolates

Antimicrobial susceptibility testing was performed according to the principles of the CLSI M43‐A guideline, with modifications optimized for *M. felis* characteristics [24, 31]. *Mycoplasma felis* isolates were cultured in liquid medium to a concentration of 10^4^ CCU/mL [32], and antibiotic standards were serially diluted twofold in *Mycoplasma* medium using 96‐well microplates, with each well then inoculated with 100 μL of the prepared *M. felis* culture. Although the CLSI guidelines recommend an incubation period of 18–24 h, considering the variability in growth rates among different *M. felis* isolates, the incubation endpoint in this study was not determined by a fixed time, but rather by the color change of the phenol red indicator in the medium (from red to yellow, indicating acid production due to *Mycoplasma* growth), with the actual incubation time varying depending on the growth rate of each isolate. A comprehensive control system was established, including growth control (wells inoculated with *M. felis* without antibiotics), medium control (uninoculated wells containing medium only), and antibiotic potency control (the reference strain *Escherichia coli* ATCC 25922 with known susceptibility). Results were considered valid only when all control conditions were met: no color change in the highest antibiotic concentration control wells (without *M. felis* inoculation), and distinct color change observed in growth control wells [32]. The minimal inhibit concentration (MIC) was defined as the lowest antibiotic concentration that completely inhibited color change. Due to the current absence of official clinical breakpoints for *M. felis*, the MIC values obtained in this study were primarily used to characterize differences in antimicrobial resistance profiles among isolates.

### 2.7. Evaluation of H_2_O_2_ Production Capacity and In Vitro Virulence Assessment of *Mycoplasma felis*

The *M. felis* isolate cultures were incubated at 37°C under 5% CO_2_ until reaching logarithmic growth phase. Three biological replicates were prepared for each strain, along with medium‐only controls. The *M. felis* cultures were centrifuged at 12,000 rpm for 15 min, and the pellets were washed once with PBS before resuspension. Glycerol was added to a final concentration of 1 mM, followed by 6‐h incubation at 37°C/5% CO_2_ with concurrent CCU determination. Postincubation, 100 μL aliquots were collected for H_2_O_2_ quantification using the Beyotime Hydrogen Peroxide Assay Kit. All data were analyzed using GraphPad Prism 7 software. Strain selection for subsequent in vivo pathogenicity studies was based on H_2_O_2_ production, and all in vitro assays comprised at least three independent experiments. The results of three independent experiments and were evaluated using ANOVA and *t*‐tests, with *p* < 0.05 considered statistically significant.

### 2.8. In Vivo Pathogenicity Evaluation of *Mycoplasma felis* Isolates Using Feline Models

Fifteen healthy female domestic cats, aged 8–12 weeks and weighing 1.0–1.5 kg, were selected for the animal experiment. All cats were obtained from a specific‐pathogen‐free (SPF) cattery in Yingcheng, Hubei Province, and were confirmed negative for *M. felis*, FHV, FCV, *C. felis*, and *Bb* by PCR using pooled respiratory swabs (conjunctival, nasal, and oropharyngeal). Their metabolic inhibition test (MIT) titers against *M. felis* were less than 1:2, and none had been vaccinated against *M. felis*. The cats were housed under uniform environmental conditions and fed 50 g of commercial feline diet daily. All experimental procedures involving cats, including animal care, euthanasia, and disposal of deceased animals, were conducted in accordance with the Ethics of Animal Care guidelines of Huazhong Agricultural University, with approval granted under Permit Number HZAUCA‐2025‐0022.

Adhering to the 3R principles (replacement, reduction, and refinement), two *M. felis* isolates exhibiting the highest in vitro virulence were selected for in vivo infection studies. Each isolate was administered intranasally at a dose of 10^8^ CCU to five British Shorthair cats per strain, with an additional group of five cats serving as unchallenged negative controls. From the day of challenge (Day 0) through Day 14 postchallenge, the following parameters were monitored daily: clinical signs, rectal temperature, respiratory disease scores (based on a standardized feline respiratory clinical assessment scale; Table 2) [33]. On Day 15 postchallenge, all animals were humanely euthanized and subjected to complete necropsy. Histopathological examination following hematoxylin and eosin (H&E) staining to assess lesion severity. Tissue samples from multiple organs (including lungs, trachea, mediastinal lymph nodes, spleen, and nasal turbinates) were collected for *M. felis*‐specific quantitative real‐time PCR (qPCR) detection to evaluate bacterial dissemination. The qPCR reaction was performed in a total volume of 20 μL, containing 2 μL of template DNA. The amplification mixture was prepared in qPCR tubes as follows: qPCR mix, for example, 10 μL 2 × qPCR Master Mix, 0.4 μL each of forward and reverse primers (10 μM), 0.2 μL probe (10 μM), 2 μL template DNA, and nuclease‐free water to adjust the final volume to 20 μL. A positive control (standard plasmid) and a negative control (2 μL ddH_2_O as template) were included in each run.

**Table 2:** Feline clinical symptom scores.

Parameter	Normal (0)	Mild (1)	Moderate (2)	Severe (3)
Rectal temp	37–39.5°C	>39.5°C	<37.0°C	≤36.0°C
Nasal/ccular discharge	Absent	Serous, mild epiphora	Mucopurulent, purulent conjunctivitis	Nasal obstruction, corneal ulceration
Cough frequency	None	<3 episodes/day	3–10 episodes/day	>10 episodes/day or dyspnea
Appetite	Normal	30%–50% reduction	>50% reduction	Anorexia >48 h

The qPCR assay was performed under the following thermal cycling conditions: Stage 1: 37°C for 2 min; Stage 2: 95°C for 5 min; Stage 3: 40 cycles of 95°C for 10 s and 59°C for 30 s. Fluorescence signals were acquired during the annealing/extension step. The primers and probe used were as follows: forward primer 5^′^‐TAAATTAGCTCTTGATGGTGTTCCT‐3^′^, reverse primer 5^′^‐TTCAAAGTCTTTTTCTGGAGTTTCA‐3^′^, and probe 5^′^‐[6‐FAM]TGAGAAGAAAAAGTTATGGAATTAATGGATGCA[BHQ1a]‐3^′^.

### 2.9. MIT for Mycoplasma Antibody (Ab)Titration

Fifteen healthy domestic cats aged 8–12 weeks, confirmed negative for *M. felis*, FHV, FCV, *C. felis*, and *Bb* by PCR and with MIT titers below 1:2, were selected for a preliminary study evaluating vaccine immunogenicity. Fifteen *M. felis* isolates were inactivated with β‐propiolactone, emulsified with Seppic Gel 02 adjuvant, and used to immunize one cat per isolate. The vaccine candidates were laboratory‐prepared inactivated whole‐cell formulations. The immunization protocol (including dose and route) was established based on preliminary experiments and optimized for this animal model. Serum samples were collected via venous puncture, and those exhibiting ELISA titers exceeding 1:2048 were selected for further analysis. The indirect ELISA method for detecting *M. felis* Abs was developed in‐house, with the following optimized parameters: optimal antigen (Ag) coating concentration of 1 mg/L, blocking with 2% BSA for 45 min, optimal serum dilution of 1:128 incubated for 45 min, optimal secondary Ab dilution of 1:5000 incubated for 45 min, and a cut‐off value of *D*_450_ = 0.352. The ELISA Ab titer was defined as the reciprocal of the highest serum dilution at which the absorbance value exceeded 2.1 times the mean value of the negative control. Detailed optimization and validation parameters of the in‐house indirect ELISA are provided in Appendix 1.

Based on the principle that specific Abs inhibit *Mycoplasma* growth and metabolism, the MIT procedures followed CLSI guideline M43‐A [31] and Chinese Pharmacopoeia (2020 Edition, Volume IV, General Chapter 3301) to determine serum Ab titers [34, 35]. Test serum samples underwent twofold serial dilution in *Mycoplasma* culture medium, followed by inoculation with standardized *Mycoplasma* suspension. After incubation: positive result: no color change in medium (indicating Ab‐mediated metabolic inhibition); negative result: color change occurred; validation control: pure *Mycoplasma* culture control tubes consistently showed metabolic color change [35]. Positive control serum: derived from cats immunized with β‐propiolactone‐inactivated *M*. *felis* isolate, exhibiting Ab titers >1:2048 as validated by *M. felis*‐specific ELISA. Negative control serum: prepared from residual serum supernatant of feline blood samples confirmed negative for respiratory pathogens via veterinary multiplex qPCR panel (GLinx Feline Respiratory Disease Profile), with absence of *M. felis* Abs confirmed by identical ELISA testing. The reaction setup, cycling conditions, and analytical sensitivity evaluation of the commercial panel are summarized in Appendix 2.

## 3. Results

### 3.1. Geographic Distribution and Epidemiological Profiling

Through testing 4329 collected feline respiratory swab samples across China, 579 *M. felis* positive samples were identified (13.37% positivity rate; Figure 1A), with coverage spanning 21 provinces where Hubei, Shanxi, Shandong, Shaanxi, Sichuan, Yunnan, Hunan, and Jiangxi each yielded over 200 samples. Notably, the high positivity rate and frequency of *M. felis* detection, coupled with the lack of systematic national epidemiological data for this pathogen in China, prompted us to focus our subsequent in‐depth analysis on *M. felis*. Guangxi (47.86%), Hebei (34.38%), Liaoning (29.08%), and Yunnan (26.91%) exhibited the highest positivity rates, while Shanxi (0.96%), Henan (4.49%), and Shanghai (4.96%) showed the lowest. These 579 positive cases were distributed across veterinary hospitals, catteries, and stray cat populations nationwide. Positive samples primarily originated from catteries in Hubei, Liaoning, Hunan, Beijing, Chongqing, Heilongjiang, and Jilin, whereas veterinary hospitals constituted the main source in other provinces, with *M. felis*‐positive specimens exclusively detected in stray cat populations in Zhejiang, Anhui, Henan, and Shanxi (Figure 1A).

Figure 1Geographic distribution and epidemiological profiling of *Mycoplasma felis* Infections in China, 2022–2024: (A) Geographic distribution of *M. felis* in China (2022–2024). Data points on the map represent positive sample counts per region, with circle color intensity (light‐to‐dark gradient) and size proportionally indicating the *M. felis* positivity rate. The legend identifies sampling locations: veterinary hospitals, catteries, and stray cat habitats. (B) Comparison of the number of positive respiratory swabs and positivity rates between healthy cats and cats with respiratory disease in different regions of China. Bar chart (left *y*‐axis) indicates the number of positive respiratory swabs, and line chart (right *y*‐axis) indicates the positivity rate of respiratory swabs. Pink bars and orange triangle lines represent healthy cats, while cyan bars and blue inverted triangle lines represent cats with respiratory disease. ns: not statistically significant;  ^∗^ *p* < 0.05,  ^∗∗^ *p* < 0.01,  ^∗∗∗^ *p* < 0.001. The horizontal axis sequentially represents North China, Northeast China, East China, Central China, South China, Southwest China, and Northwest China. (C) Pathogen distribution among *M. felis*‐positive samples (2022–2024). Bar heights indicate detection frequencies of respiratory pathogens: FHV, FCV, *M. felis*, *C.felis*, and *Bb*. Overlaid scatter points denote individual sample distributions, with error bars (horizontal lines) showing data range.

**Figure:**
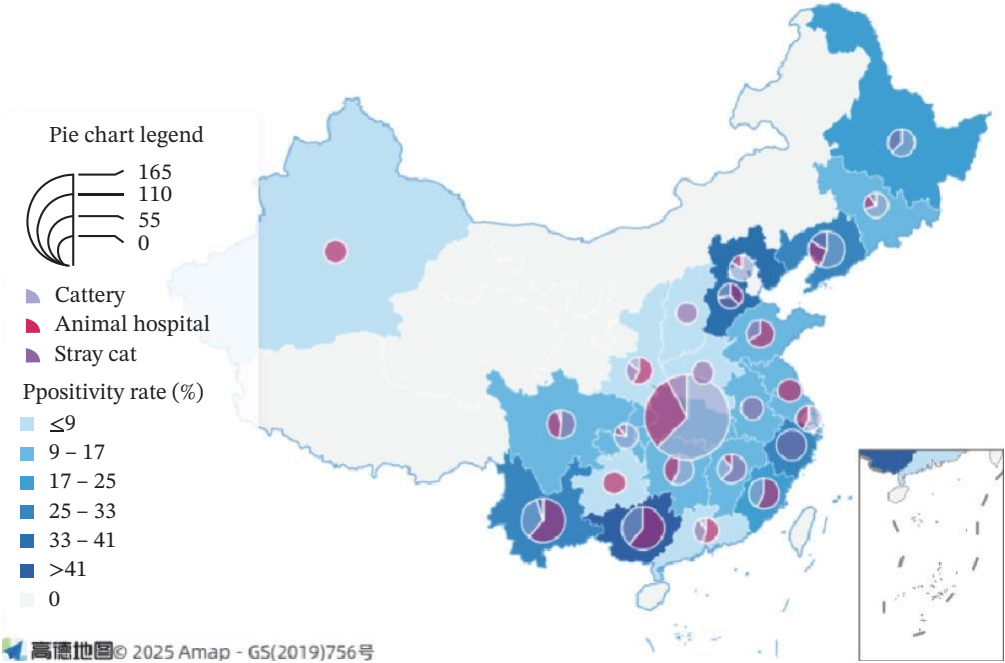
(A)

**Figure:**
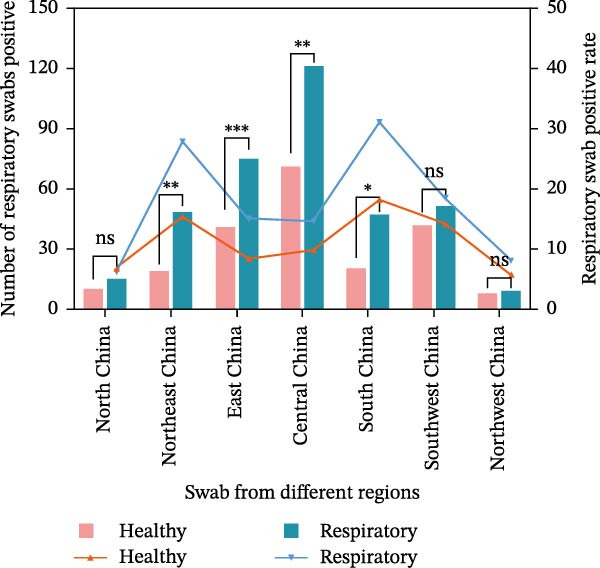
(B)

**Figure:**
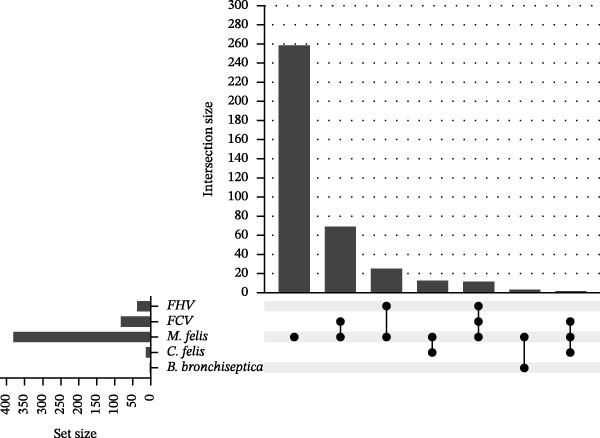
(C)

*Mycoplasma* detection across seven regions of China revealed higher overall positivity rates and case numbers in groups with respiratory symptoms than in healthy controls, with marked interregional variability. Central China showed the highest absolute number of positive cases and a significantly elevated positivity rate in the symptomatic group (*p* < 0.01); Eastern China exhibited an even stronger association (*p* < 0.001). Significant differences were also observed in Northeastern (*p* < 0.01) and Southern China (*p* < 0.05). In contrast, no significant differences were found in Northern, Southwestern, or Northwestern China (*p* > 0.05). Positivity rates among symptomatic groups peaked in Northeastern and Southern China. Although Central China had the highest case count, its positivity rate advantage was slightly lower than that of Southern China, likely reflecting differences in sample size. These findings demonstrate distinct geographical heterogeneity in *Mycoplasma* infection across China, with particularly strong correlations with respiratory symptoms in Eastern and Central China (Figure 1B).

Among 382 *M. felis*‐positive samples from cats exhibiting respiratory symptoms (Figure 1C), PCR–based detection of FHV, FCV, *M. felis*, *C.felis*, and *Bb* revealed mono‐infection predominance (67.8%, 259/382), with *M. felis* + FCV being the most frequent combination (18.3%, 70/382), followed by *M. felis* + FHV (6.0%, 23/382). Triple‐pathogen infections represented 3.4% (13/382), primarily comprising *M. felis* + FCV + FHV combinations.

### 3.2. Phylogenetic Analysis of *16S rRNA* Gene and Morphological Characterization of Mycoplasma Isolates From Cats

Through isolation and cultivation of 259 *M. felis* infection samples confirmed by etiological diagnosis, we successfully obtained 15 representative strains (Figure 2A). Phylogenetic analysis based on *16S rRNA* gene sequencing revealed two distinct evolutionary branches among respiratory infection strains, exhibiting significant differences in in vitro growth requirements: Branch I strains (e.g., *M. felis*‐9) formed characteristic fried‐egg colonies (30–160 μm in diameter) on standard PPLO agar and displayed pleomorphic morphology under electron microscopy; whereas Branch II strains (*M. felis*‐6, ‐8, ‐25) identified as a novel branch phylogenetically distant from known *M. felis*‐required amino acid–supplemented medium for growth, forming unique Morella‐like microcolonies with predominantly spherical ultrastructure (Figure 2B,C). Transmission electron microscopy (TEM) confirmed that *M. felis*‐8 exhibited morphological features highly similar to *Morus* spp. fruits, particularly spherical surface protrusions (120–150 nm in diameter) and granular textures (Figure 2D,E). Both branch types were identified as *M. felis* by Dienes staining (Figure 2F,G). Therefore, in this study, we retained the original strain nomenclature while provisionally designating the fried‐egg colony morphology (Branch I strains) as the Myco type and the Morella‐like morphology (Branch II strains) as the Morella type based on phenotypic characteristics. Additionally, phylogenetic analysis revealed that *M. felis*‐29 clustered neither with these two types, but instead formed a distinct clade with *M. gateae*, indicating high genetic affinity.

Figure 2Phylogenetic analysis of *16S rRNA* gene and morphological characterization of *Mycoplasma* Isolates from cats: (A) Phylogenetic tree of *16S rRNA* gene sequences from Chinese *Mycoplasma* isolates, illustrating genetic relationships among strains from diverse hosts (cats, cattle, goats, etc.) and sources. Branch colors denote origin (NCBI reference sequences vs. clinical isolates), while node shapes indicate culture conditions. (B) Natural *M. felis*‐28 colony morphology under low magnification (40x). (C) Natural *M. felis*‐8 colony morphology under low magnification (40x). (D) Transmission electron micrographs demonstrating *M. felis*‐28 structure (scale bars: 100 nm). (E) Transmission electron micrographs demonstrating *M. felis*‐8 structure (scale bars: 100 nm). (F) Dienes‐stained *M. felis*‐28 organisms at high magnification (100x oil immersion). (G) Dienes‐stained *M. felis*‐8 organisms at high magnification (100x oil immersion).

**Figure:**
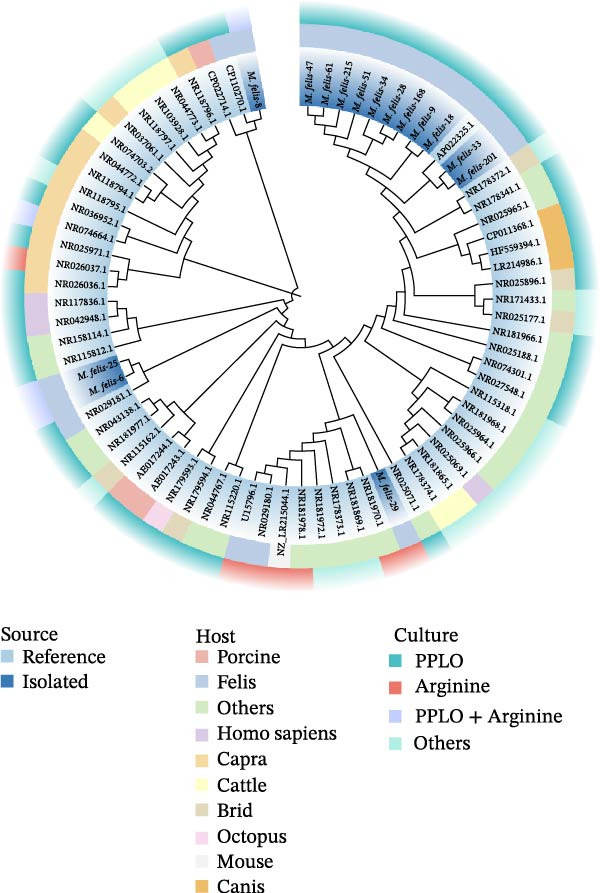
(A)

**Figure:**
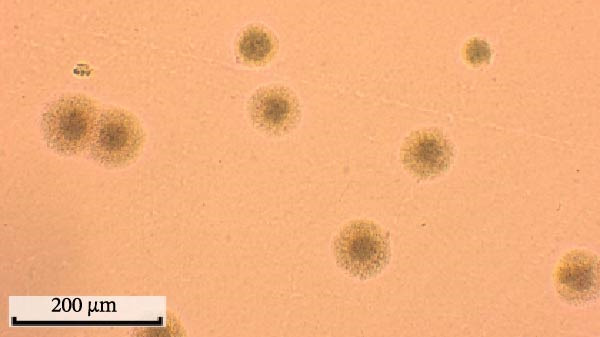
(B)

**Figure:**
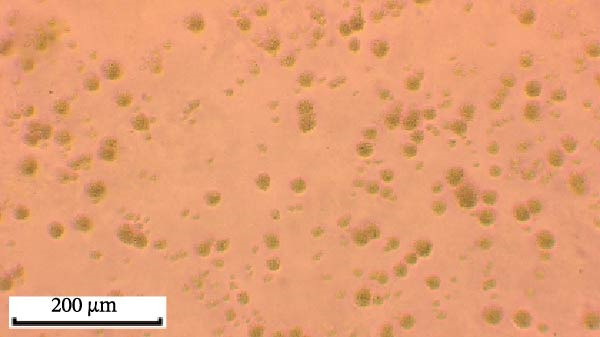
(C)

**Figure:**
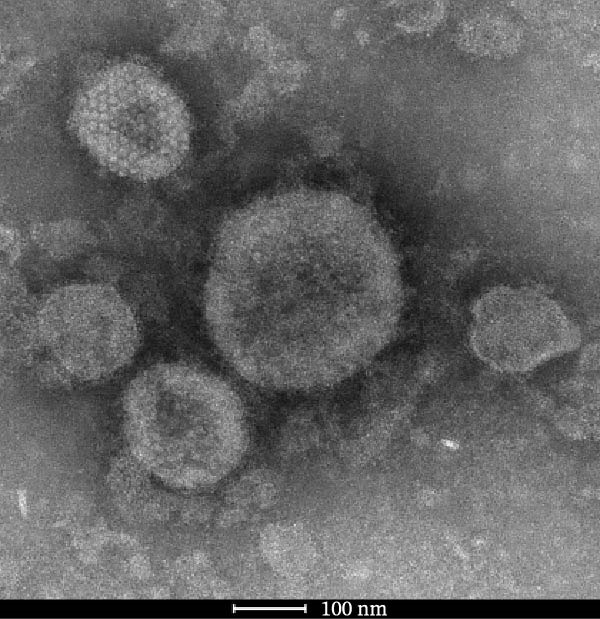
(D)

**Figure:**
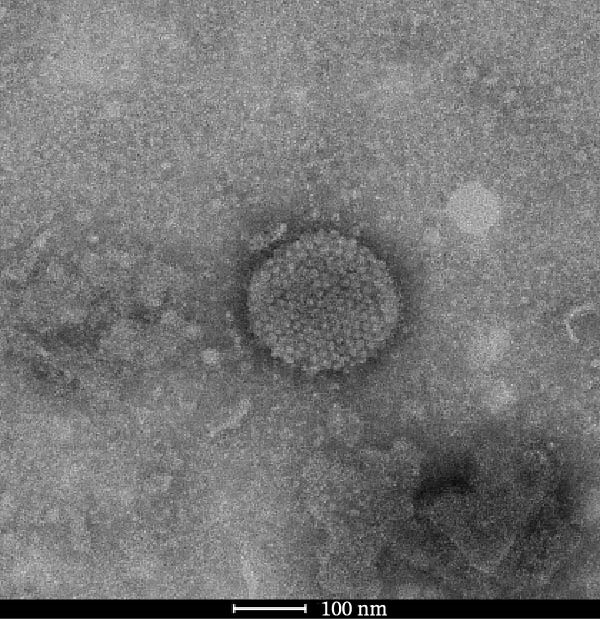
(E)

**Figure:**
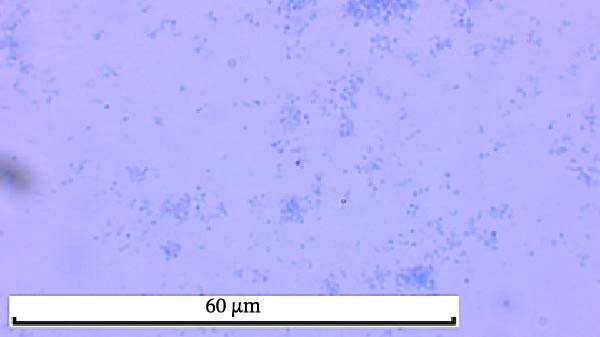
(F)

**Figure:**
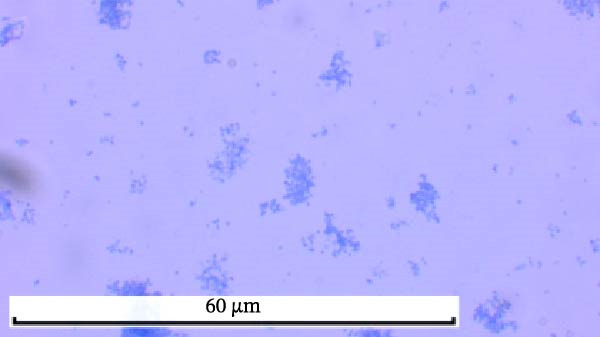
(G)

### 3.3. Cluster Analysis of Proliferation Dynamics and pH Adaptability in *Mycoplasma felis* Strains

Fifteen feline *Mycoplasma* strains were cultured to reveal strain‐specific proliferation patterns: the majority of isolates reached peak growth at 24–36 h, while *M. felis*‐28/29/33 exhibited differential growth kinetics characterized by a prolonged logarithmic phase extending beyond 36 h. Cluster analysis delineated three major groups (Figure 3A), with *M. felis*‐28/29/33 forming a distinct cluster demonstrating delayed entry into logarithmic phase and attenuated growth; *M. felis*‐6/9/18/34/215 constituting a rapidly proliferating yet accelerated‐decline group; and the remaining strains showing moderate growth with gradual decline. Concurrent monitoring of pH dynamics during proliferation (Figure 3B) revealed metabolic divergence among isolates: most strains induced significant acidification, achieving peak growth at pH 5.5–6.6, though *M. felis*‐33 displayed markedly reduced acidification. Notably, *M. felis*‐29 diverged through alkalinization (pH elevation >1.5 units) via arginine‐dependent deamination, exhibiting high similarity to *M. gateae*. The Morella‐type strains *M. felis*‐6/8/25 coclustered within a subbranch, suggesting convergent metabolic adaptation.

Figure 3Cluster analysis of proliferation dynamics and pH adaptability in *Mycoplasma felis* strains: (A) Radial plot illustrating proliferation kinetics of *Mycoplasma* strains using color‐coded phylogenetic clusters (Morella: red; Myco: blue; *gateae*: gray) and concentric rings denoting CCU/mL values at sequential timepoints. (B) Radial visualization of pH‐dependent growth profiles. Colors represent taxonomic groups (as in Subpart (A)), with rings indicating real‐time pH changes during incubation.

**Figure:**
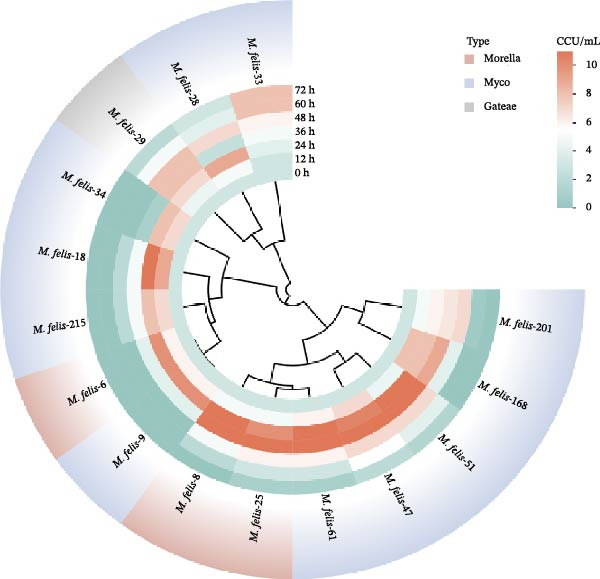
(A)

**Figure:**
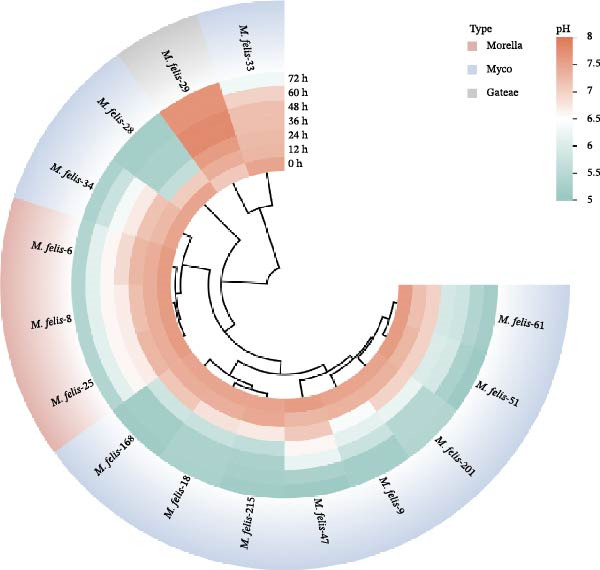
(B)

### 3.4. Antimicrobial Susceptibility Profiling and Diversity of *Mycoplasma felis* Clinical Isolates

**Figure 4:**
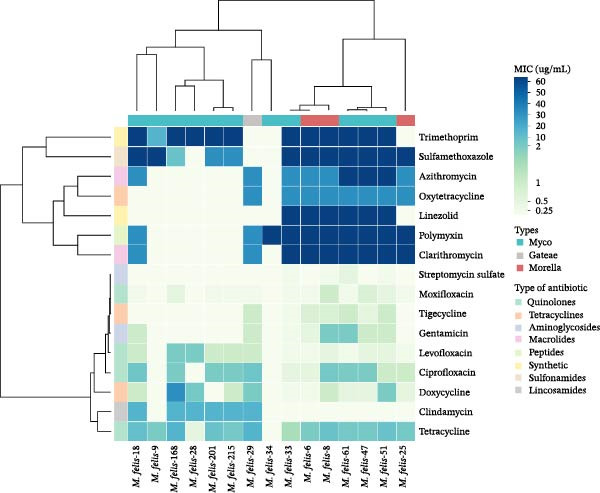
Heatmap analysis of minimum inhibitory concentrations (MICs) for antimicrobial susceptibility in *Mycoplasma felis* Isolates. The top dendrogram illustrates the genetic relationships among 15 feline *Mycoplasma* isolates, while the left dendrogram clusters antibiotics based on similar inhibitory profiles against these strains. In the heatmap, color intensity correlates with MIC values (μg/mL), where deeper hues indicate higher MICs (i.e., reduced efficacy). The top color bar categorizes strains into morphotypes (Myco, Gateae, Morella), and the left color bar denotes antibiotic classes (e.g., quinolones, tetracyclines, aminoglycosides).

Antimicrobial susceptibility testing of 15 *M. felis* isolates revealed considerable variability in MIC values across different antimicrobial classes. For fluoroquinolones, levofloxacin MICs ranged from 0.125 to 2 μg/mL, moxifloxacin from 0.125 to 1 μg/mL, and ciprofloxacin from 0.125 to 8 μg/mL. Tetracyclines exhibited broad MIC ranges: tetracycline 0.125–16 μg/mL, doxycycline 0.125–32 μg/mL, and oxytetracycline 0.125–32 μg/mL, with a distinct bimodal distribution (most isolates either highly susceptible at 0.125 μg/mL or resistant at 32 μg/mL). Notably, one isolate (*M. felis*‐168) showed a doxycycline MIC of 32 μg/mL, suggesting potential resistance. Tigecycline was uniformly active, with MICs ≤1 μg/mL for all strains. Among aminoglycosides, streptomycin sulfate demonstrated the highest potency, with MICs ≤0.5 μg/mL (mostly 0.125 μg/mL), while gentamicin MICs ranged from 0.125 to 2 μg/mL. Macrolides displayed a clear dichotomy: azithromycin and clarithromycin MICs were ≤0.125 μg/mL in susceptible isolates but ≥32 μg/mL in eight of 15 (53.3%) strains, indicating widespread macrolide resistance. Clindamycin MICs varied from 0.125 to 16 μg/mL, with several strains exhibiting elevated values (16 μg/mL; Figure 4). Hierarchical clustering of the susceptibility profiles divided the 15 strains into two major groups; although no significant divergence was observed between Morella‐type and Myco‐type *M. felis*, the three Morella‐type strains (isolates *M. felis*‐6, *M. felis*‐8, and *M. felis*‐25) clustered together, sharing similar drug susceptibility patterns.

### 3.5. Virulence Traits of Feline Mycoplasma In Vitro and Vivo

The capacity for H_2_O_2_ production among 15 strains of feline *Mycoplasma*s demonstrated that strain *M. felis*‐29, belonging to the *M. gateae* cluster, exhibited the lowest levels. In contrast, strains *M. felis*‐8 and *M. felis*‐28 were identified as the most potent producers within the Morella and Myco groups, respectively. Significant variability in H_2_O_2_ production was observed among individual strains within both the Morella and Myco groups, without any discernible correlation (Figure 5A).

Figure 5In vitro/vivo virulence assessment of *M. felis*: hydrogen peroxide production and histopathological alterations. (A) Hydrogen peroxide (H_2_O_2_) production capacity of *M. felis* strains in vitro. Data are expressed as micromoles (μM) per 10^8^ CCU. Bar heights indicate strain‐specific H_2_O_2_ yields, a key reactive oxygen species (ROS) biomarker reflecting virulence potential. (B) Daily clinical scores in *Mycoplasma*‐challenged cats throughout the 14‐day trial. (C) Rectal temperature monitoring in *Mycoplasma*‐challenged cats throughout the 14‐day trial. (D) Relative DNA detected by qPCR loads of *M. felis*‐8 and *M. felis*‐28 in different organs. Bar chart showing the relative DNA levels (log‐transformed copies/μL) of *M. felis*‐8 (blue) and *M. felis*‐28 (pink) in eyelid, trachea, lung, kidney, lymph nodes, and spleen. (E) Representative histopathological changes in pulmonary, tracheal, and palpebral conjunctival tissues from infected cats.

**Figure:**
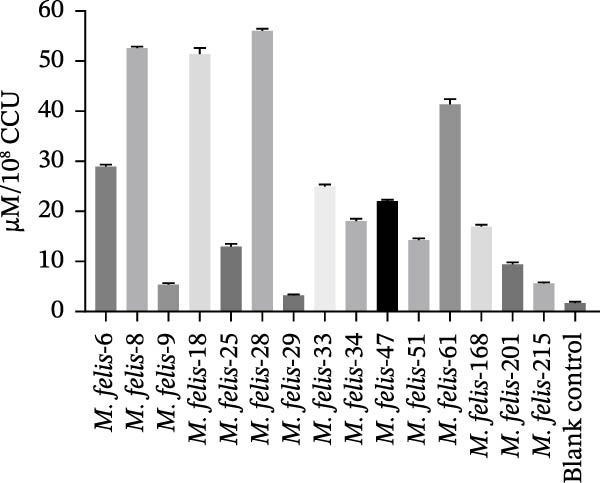
(A)

**Figure:**
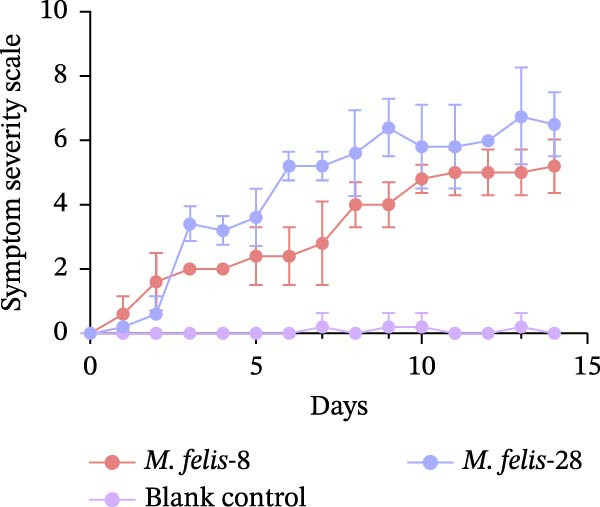
(B)

**Figure:**
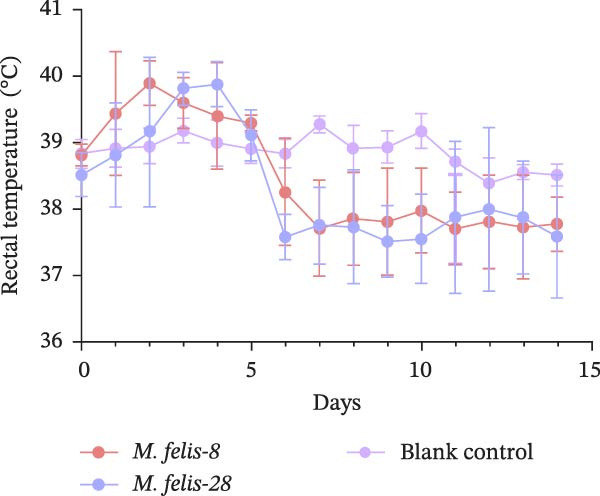
(C)

**Figure:**
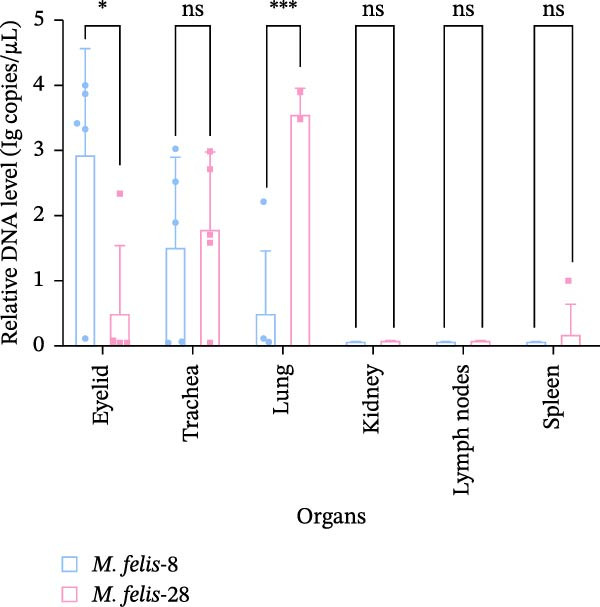
(D)

**Figure:**
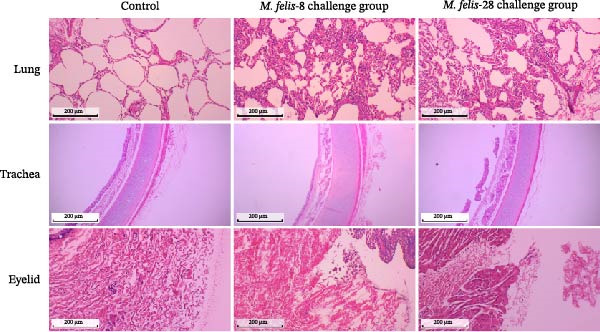
(E)

This study selected strains *M. felis*‐8 and *M. felis*‐28 from the Morella and Myco groups, respectively, based on their capacity for in vitro metabolic production of H_2_O_2_. As the two isolates exhibiting the highest H_2_O_2_ yields among all tested strains, they were chosen as representative strains for subsequent feline infection experiments. Due to constraints imposed by the 3R principles (replacement, reduction, and refinement), large‐scale in vivo challenge tests in cats were not feasible. Accordingly, the aforementioned in vitro virulence‐based screening strategy was adopted to minimize animal usage while ensuring representativeness for comparative virulence studies. No statistically significant difference in rectal temperature was observed between the Strain‐8 and Strain‐28 infection groups. However, both infection groups induced significant alterations in rectal temperature compared with the blank control group (PBS group). Clinical manifestations were assessed through scoring systems, which revealed that strain *M. felis*‐28 of the Myco group presented with more pronounced clinical signs of disease (Figure 5B,C).

Histopathological analysis of the lung tissues from the infected groups revealed more distinct areas of inflammation characterized by a dense infiltrate of inflammatory cells, potentially including lymphocytes, macrophages, and plasma cells. The tissue architecture was further compromised, with an increase in interstitial space. Notably, the strain *M. felis*‐28 exhibited a particularly marked infiltration of inflammatory cells in the lungs, with the tissue structure being nearly completely disrupted. The tracheal mucosa in the infected groups displayed cellular and tissue sloughing. Histological sections of the eyelids from the infected groups demonstrated the presence of a small amount of red blood cells and inflammatory exudates within the interstitial spaces, with strain *M. felis*‐8 causing significant tissue damage, cellular injury, and detachment in the eyelid tissues (Figure 5E).

The qPCR results revealed distinct tissue tropism between the two strains, as reflected by their relative DNA loads in various organs (Figure 5D). In the eyelid, *M. felis*‐8 exhibited a significantly higher relative DNA level than *M. felis*‐28 (~3–4 log copies/μL vs. 1 log copies/μL; *p* < 0.05). In the trachea, although *M. felis*‐8 showed a slightly higher DNA level than *M. felis*‐28, the difference was not statistically significant (*p* > 0.05). In contrast, in the lung, *M. felis*‐28 displayed a markedly higher DNA load compared to *M. felis*‐8 (~4–5 log copies/μL vs. 1–2 log copies/μL; *p* < 0.001). In the kidney, lymph nodes, and spleen, both strains exhibited minimal DNA loads, with no significant differences observed (*p* > 0.05). Collectively, these findings indicate that *M. felis*‐8 predominantly colonizes the upper respiratory tract, particularly the eyelid, whereas *M. felis*‐28 exhibits a pronounced tropism for the lower respiratory tract, specifically the lung. This distribution pattern is highly consistent with the histopathological lesion patterns observed in the corresponding tissues.

### 3.6. Analysis of the Metabolic Inhibition Effect and Cross‐Reactivity of Abs and Ags of *Mycoplasma felis*

**Figure 6:**
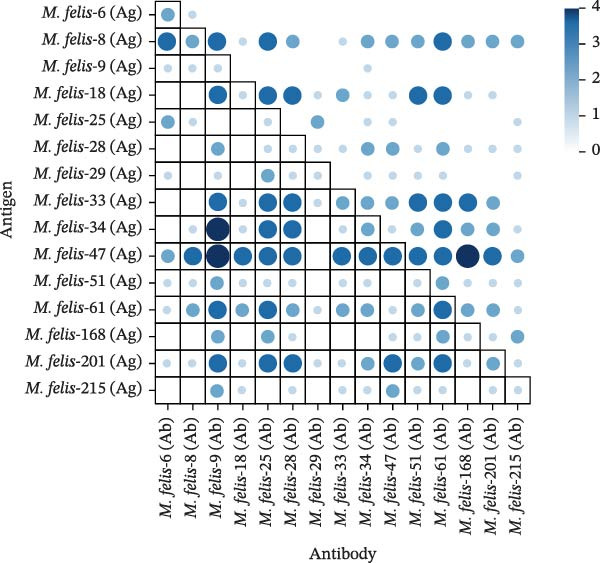
Cross‐validation heatmap of antibody–antigen metabolic inhibition for 15 *M. felis* strains. This heatmap visually presents the cross‐validation results of metabolic inhibition tests (MIT) between antibodies and antigens from 15 *M. felis* strains. Rows: Represent different *M. felis* antigen preparations (e.g., *M. felis*‐6 (Ag) and *M. felis*‐8 (Ag)); columns: represent positive sera from different *M. felis* strains (e.g., *M. felis*‐6 (Ab) and *M. felis*‐8 (Ab)); color gradient: a light‑to‑dark blue gradient indicates increasing metabolic inhibition intensity, darker colors signify stronger inhibitory effects; circle size: proportional to inhibition potency (largest diameter = titer ≥1:16); color scale: right‐side legend displays log_2_‐transformed inhibition titers (*n*), corresponding to antibody dilution series (1:2^*n*^), *n* = 0: baseline inhibition (1:1), higher *n* values indicate greater inhibition efficacy.

Cross‐protection analysis revealed significant Ag–Ab cross‐reactivity among feline *M. felis* strains: *M. felis*‐47 and *M. felis*‐61 exhibited high homologous reactivity (Ag–Ab autologous pairs) with titers of 1:8; *M. felis*‐47 Ag demonstrated pronounced reactivity with heterologous Abs (except against *M. felis*‐29 Ab), showing peak titers of 1:16 with *M. felis*‐9 Ab and *M. felis*‐168 Ab; significant cross‐reactivity was likewise observed between *M. felis*‐34 Ag and *M. felis*‐9 Ab (titer 1:16). Conversely, *M. felis*‐6 Ag showed minimal reactivity with most Abs (titers ≤1:2). *M. felis*‐9 Ab exhibited broad cross‐reactivity against multiple Ags (majority titers ≥1:4), while *M. felis*‐29 Ab demonstrated negligible reactivity toward most Ags (Figure 6).

## 4. Discussion

This study delineates the inaugural comprehensive epidemiological landscape of feline *Mycoplasma* infections across China, demonstrating an aggregate prevalence of 13.37% (579/4329 cases) with significant regional heterogeneity. Elevated detection rates in southwestern China (e.g., Guangxi and Yunnan) reflect humidity‐mediated pathogen persistence, whereas cattery‐associated outbreaks in northern provinces (Heilongjiang, Jilin, and Liaoning) are attributable to population density—the predominant determinant of high positivity rates in intensive husbandry systems. The heightened infection burden within cattery populations necessitates accelerated development of prophylactic and therapeutic countermeasures. Notably, molecular detection of *M. felis* in Central China (Zhejiang, Anhui, Henan, and Shanxi) demonstrated exclusive restriction to stray cat populations. This epidemiologic reservoir warrants imperative vigilance considering China’s 19% expansion in stray feline demographics (CAWS, 2024), establishing novel anthropozoonotic transmission vectors congruent with documented zoonotic capacities of *M. felis* [17]. Furthermore, a recent surveillance study in Shanghai reported an *M. felis* positivity rate of 18.72% (70/374) in urban stray cats, with the pathogen detected year‐round and across multiple seasons [20], indicating that clinically healthy stray cats can serve as asymptomatic carriers and play a significant role in the maintenance and transmission of *M. felis* within feline populations [20].Unexpectedly, 67.8% of *M. felis*‐positive cases represented mono‐infections, challenging the conventional view of *Mycoplasmas* as opportunistic secondary pathogens [36]. The frequent coinfection with FCV (18.3%) suggests synergistic pathogenesis, consistent with Australian reports detecting FCV and *M. felis* as copathogens in 13% (*n* = 205/1533) of feline URTI cases [17]. *Mycoplasma felis* infections exhibited significant heterogeneity across different regions. The association between infection and respiratory symptoms was most pronounced in East China and Central China, underscoring the clinical significance of this pathogen in these areas. Although statistically significant differences were also observed in Northeast and South China, the degree of association varied. In contrast, no significant differences were found in North, Northwest, or Southwest China, which may be attributed to variations in sample size or husbandry practices [19].

Clinical studies on feline *Mycoplasma* isolates remain limited, and current multiplex qPCR assays for feline respiratory pathogens cannot effectively subtype *M. felis* [27]. Molecular characterization of corneal scrapings derived from seven feline subjects presenting ulcerative keratitis and keratomalacia yielded definitive etiological attribution to six *M. felis* strains and one *M. gateae* isolate through *16S rRNA* gene amplification, as documented by Gray et al. [12]. *Mycoplasma gateae*, a species infrequently documented in feline respiratory pathologies but principally linked to arthritis and tenosynovitis, was isolated as a singular occurrence in this investigation. In contrast, the 14 *M. felis* strains underwent selection predicated on geographical distinctiveness and intraspecies nucleotide divergence exceeding 0.5% within *16S rRNA* gene loci, consistent with established taxonomic criteria [11, 12]. Notably, one representative isolate (Brach II) identified in this study exhibited unique nutritional dependency during in vitro culture, along with Morella colony morphology. Critical culture experiments revealed that this strain could only achieve stable passage and clonal purification in medium supplemented with specific amino acids; in basal medium lacking these amino acid supplements, the strain could not be continuously cultured in vitro. This phenomenon suggests that this isolate may possess specific amino acid auxotrophic characteristics, with its growth and metabolism dependent on certain exogenous amino acids. Although previous studies have demonstrated significant differences in amino acid metabolic pathways among different *Mycoplasma* species, reports on amino acid nutritional dependency at the strain level of *M. felis* remain limited. This study reports, for the first time, the discovery of such *M. felis* strains with unique [37] nutritional dependencies during epidemiological investigation and provides preliminary observations on their basic culture characteristics. However, the precise amino acid requirements, underlying metabolic mechanisms, and potential biological significance of this strain warrant further in‐depth investigation. Integrative analysis of epidemiologically relevant isolates through high‐throughput sequencing, complemented by phylogenetic reconstruction, in vitro culture and colony morphotyping, delineated two novel clades within *M. felis*: the Myco‐type, characterized by classical fried‐egg‐like colonial architecture, and the Morella‐type, exhibiting distinctive mulberry‐structured microcolonies. The dual characteristics of auxotrophy for amino acid–enriched media and ultrastructural congruence with *M. gateae* observed in the Morella clade collectively indicate adaptive genomic minimization. This evolutionary strategy enables persistence within nutrient‐constrained mucosal ecosystems through speciation‐driven metabolic divergence, consistent with established *Mycoplasma* evolutionary paradigms [38]. Metabolic profiling revealed clade‐specific strategies: both Myco and Morella strains acidified media via glucose fermentation, whereas *M. gateae*‐like isolates (e.g., *M. felis*‐29) exhibited suspected weak alkalinization through arginine deamination.

Notwithstanding the paucity of clinical evidence regarding antimicrobial resistance in feline *Mycoplasmas* [39], MIC findings in this research elucidate emergent resistance trends. *Mycoplasma* species are generally considered susceptible to macrolides, tetracyclines, fluoroquinolones, lincosamides, and aminoglycosides, but intrinsically resistant to β‐lactams due to their cell wall‐deficient nature [24, 32, 38]. Our antimicrobial susceptibility profile reveals that macrolide susceptibility exhibits a clade‐dependent divergence (Morella > *gateae* > Myco), characterized by a pronounced bimodal MIC distribution. The emergence of doxycycline‐resistant strains (*M. felis*‐168 MIC = 32 μg/mL) signals diminished efficacy of first‐line therapeutics, likely driven by agricultural tetracycline misuse [6]. Universal fluoroquinolone sensitivity (MIC <0.25 μg/mL) supports empirical use, though escalating veterinary fluoroquinolone consumption warrants vigilance. Consequently, evidence‐based stewardship frameworks necessitate integration of antimicrobial susceptibility surveillance with empirical prescribing paradigms. Specifically, implementing colonial morphotyping on primary isolates is advocated‐whereby detection of mulberry‐structured microcolonies indicates deployment of spectrum‐tailored anti‐*Mycoplasmal* agents targeting Morella‐type phenotypes, per CLSI VET08 guidelines. Morella‐type conjunctivitis requires doxycycline as primary therapy with levofloxacin as alternative [6]; Myco‐type pneumonia necessitates levofloxacin as first‐line with tigecycline as backup agent. Azithromycin should be contraindicated as first‐line therapy due to established resistance determinants [40] and this study. In scenarios where *Mycoplasma* culture isolation and subsequent morphotyping remain inaccessible, streptomycin sulfate constitutes a validated broad‐spectrum alternative with demonstrated in vitro efficacy against diverse *Mycoplasma* phenotypes.

H_2_O_2_ production was identified as a key in vitro virulence factor (*R* ^2^ = 0.87, *p*  < 0.001) [41]. Highly virulent strains *M. felis*‐8 (Morella type) and *M. felis*‐28 (Myco type) produced H_2_O_2_ at concentrations of 52–57 μM/10^8^ CCU, a level sufficient to elicit significant oxidative DNA damage and ciliary paralysis in ex vivo feline tracheal explants [42]. This peroxide‐mediated cytotoxicity explains the histopathological findings: pulmonary tropism triggers alveolar hemorrhage and neutrophil infiltration through ROS‐mediated endothelial damage [2].

*Mycoplasma* species have been implicated as etiological agents of feline conjunctivitis, lower respiratory tract infections, and polyarthritis [18, 43]. However, substantial evidence indicate that *M. felis* can cause feline upper respiratory tract infections [12, 44]. While seemingly contradictory, this study discovered differences in tissue tropism between *M. felis*‐8 (Morella‐type) and *M. felis*‐28 (Myco‐type), with *M. felis*‐8 and *M. felis*‐28 primarily infecting the upper and lower respiratory tracts, respectively. Raspberry‐like adhesins enhance binding to conjunctival epithelium, exacerbating damage, reflecting clade‐specific tissue adaptation; the spherical surface projections of the Morella‐type *M. felis* promote binding to corneal mucins [41].

Weak Ab cross‐reactivity exists between the Myco and Morella clades, suggesting the potential for developing an *M. felis* vaccine [1]. The high autoreactivity of autoantigen Abs and [17] conserved antigenic epitope regions. These could serve as potential universal immune targets, enabling the development of vaccines based on these [45] strains to induce more stable and broadly protective immunity. Weakly reactive combinations (e.g., *M. felis*‐6 (Ag) with most Abs and *M. felis*‐29 (Ab) with most Ags) reflect the antigenic uniqueness of some strains. These low‐cross‐reactivity “strain‐specific” isolates represent promising candidates for developing diagnostics with enhanced analytical specificity, thereby eliminating false positives resulting from cross‐reactivity [46]. This substantiate interpretations of explaining the underdiagnosis of some infections in clinical practice.

Several limitations of this study should be acknowledged. First, regarding the conclusion on strain virulence, cautious interpretation is warranted. Although clinical symptom scores indicated that cats infected with *M. felis*‐28 exhibited more pronounced clinical manifestations during the observation period, no statistically significant difference in rectal temperature was observed between the two infected groups, suggesting that virulence assessment should integrate multiple parameters. Second, attempts to localize *M. felis* in tissues using dienes staining were hampered by technical instability in tissue sections compared to culture preparations, precluding definitive histological confirmation of bacterial distribution; nevertheless, lesion severity was generally comparable among infected individuals due to stringent preselection of experimental animals (consistent age, breed, housing conditions, and absence of common exogenous viral infections). Third, owing to the current lack of a universally accepted reference strain for *M. felis* in such infection models, this study employed a PBS control as baseline reference and focused on comparative analyses among field isolates, without inclusion of a prototype strain as external calibrator—a common practice in research on emerging pathogens. These limitations underscore the need for future investigations encompassing optimization of tissue staining techniques, establishment of standardized reference systems, and multicenter validation studies.

## 5. Conclusion

This study demonstrated that *M. felis* infects 13.37% of Chinese cats, with two novel clades identified: Myco‐type (fried‐egg colonies; lung tropism) and Morella‐type (mulberry‐like colonies; conjunctival tropism). Colonial morphology directly informs targeted therapy: levofloxacin for Myco‐type pneumonia and doxycycline for Morella‐type conjunctivitis. The emergence of macrolide resistance underscores the imperative for reinforced clinical deployment and stewardship of fluoroquinolones. This investigation bridges a critical knowledge gap in the nationwide epidemiology of *M. felis*, while characterization of two novel clades reveals substantial potential for polyvalent vaccine development.

## Author Contributions

Conceptualization: Shengbo Cao, Zijun Ye, and Congrong Wang. Methodology, investigation: Zijun Ye, Congrong Wang, Quanhui Yan, Weihui Li, Mingjun Ye, Luying Li and Yan Zhang. Validation, data curation: Zijun Ye, Quanhui Yan, and Congrong Wang. Writing – original draft preparation: Zijun Ye and Congrong Wang. Writing – review and editing: Shengbo Cao, Zijun Ye, and Congrong Wang. Supervision, project administration: Shengbo Cao. Funding acquisition: Shengbo Cao and Qiuyan Li.

## Funding

This study was supported by the National Key Research and Development Program of China (Grant 2023YFD1800302), the Hubei Provincial Laboratory Animal Research Project (Grant 2023CFA005), the Hubei Special Project for Science Development (Grant 2024CSA0600), and the Wuhan Organization Department Engineering Doctoral Industry‐Academia Collaborative Program.

## Disclosure

All authors have read and agreed to the published version of the manuscript. The funders had no role in the study design, data collection and analysis, interpretation of results, decision to publish, or preparation of the manuscript.

## Conflicts of Interest

The authors declare no conflicts of interest.

## Data Availability

All the data are contained within the article.

## Appendix 1

Validation of indirect ELISA for *Mycoplasma felis* antibody detection.

The indirect ELISA method for detecting *M. felis* antibodies was established in‐house, with optimized parameters and validation indicators as follows:

– Antigen coating concentration: Optimal coating concentration was 1 mg/L.
– Blocking conditions: Blocking with 2% BSA at 37°C for 45 min.
– Serum dilution and incubation time: Optimal serum dilution was 1:128, incubated at 37°C for 45 min.
– Secondary antibody dilution and incubation time: Optimal HRP–conjugated secondary antibody dilution was 1:5,000, incubated at 37°C for 45 min.
– Cut‐off value determination: The cut‐off value was calculated as *D*_450_ = 0.352, based on the mean *D*_450_ value of 61 known negative sera plus twice the standard deviation.
– Titer determination criteria: The ELISA antibody titer was defined as the reciprocal of the highest serum dilution at which the absorbance value exceeded 2.1 times the mean value of the negative control.

## Appendix 2

Reaction system and conditions for commercial quintuple qPCR assay.

The GLinx feline respiratory disease profile commercial qPCR kit was used for detection of feline respiratory pathogens. The optimized reaction system and cycling parameters were as follows:

– Reaction system (total volume 50 μL):
  – Primer mix (four pairs of forward and reverse primers): 1 μL each
  – FCV cDNA template: 1 μL
  – FHV‐1, *Mycoplasma felis*, *Chlamydia felis*, and Bordetella bronchiseptica (*Bb*) DNA templates: 1 μL each
  – GoldStar mix (green): supplemented to 50 μL
– Amplification conditions:
  – Annealing temperature optimization range: 51.7–60°C, with optimal annealing temperature determined as 60°C (providing the clearest target bands)
  – Cycling parameters: 95°C for 5 min (initial denaturation); 40 cycles of 95°C for 15 s and 60°C for 30 s (fluorescence acquisition)
– Sensitivity validation:
  – Minimum detectable concentration for FCV target gene: 1 × 10^−5^ ng/μL
  – Minimum detectable concentration for FHV‐1 target gene: 1 × 10^−4^ ng/μL
  – Minimum detectable concentration for *M. felis* target gene: 1 × 10^−3^ ng/μL
  – Minimum detectable concentration for *C. felis* target gene: 1 × 10^−4^ ng/μL
  – Minimum detectable concentration for *Bb* target gene: 1 × 10^−4^ ng/μL
